# Trial Design in Critical Care Nutrition: The Past, Present and Future

**DOI:** 10.3390/nu12123694

**Published:** 2020-11-30

**Authors:** Lee-anne S. Chapple, Emma J. Ridley, Marianne J. Chapman

**Affiliations:** 1 Intensive Care Unit, Royal Adelaide Hospital, Adelaide, SA 5000, Australia; marianne.chapman@sa.gov.au; 2 Discipline of Acute Care Medicine, The University of Adelaide, Adelaide, SA 5005, Australia; 3 Centre of Research Excellence in Translating Nutritional Science to Good Health, The University of Adelaide, Adelaide, SA 5005, Australia; 4 Australian and New Zealand Intensive Care Research Centre, School of Public Health and Preventative Medicine, Monash University, Melbourne, VIC 3004, Australia; emma.ridley@monash.edu; 5 Nutrition Department, Alfred Health, Melbourne, VIC 3004, Australia

**Keywords:** randomised controlled trial, intensive care, nutrition, study design

## Abstract

The specialty of nutrition in critical care is relatively modern, and accordingly, trial design has progressed over recent decades. In the past, small observational and physiological studies evolved to become small single-centre comparative trials, but these had significant limitations by today’s standards. Power calculations were often not undertaken, outcomes were not specified a priori, and blinding and randomisation were not always rigorous. These trials have been superseded by larger, more carefully designed and conducted multi-centre trials. Progress in trial conduct has been facilitated by a greater understanding of statistical concepts and methodological design. In addition, larger numbers of potential study participants and increased access to funding support trials able to detect smaller differences in outcomes. This narrative review outlines why critical care nutrition research is unique and includes a historical critique of trial design to provide readers with an understanding of how and why things have changed. This review focuses on study methodology, population group, intervention, and outcomes, with a discussion as to how these factors have evolved, and concludes with an insight into what we believe trial design may look like in the future. This will provide perspective on the translation of the critical care nutrition literature into clinical practice.

## 1. Challenges of Critical Care Nutrition Research

Artificial nutrition is routinely provided to the majority of mechanically ventilated adult patients admitted to the intensive care unit (ICU). Despite this, we have an incomplete understanding about the optimal route, timing, and dose to provide. While there are published international guidelines from Canada, the United States, and Europe [1,2,3], these are largely based on expert opinion or observational data. For example, the American Society of Parenteral and Enteral Nutrition 2016 Critical Care Guidelines provide 95 individual recommendations, of which just three are based on high or moderate-to-high quality evidence (i.e., a high-quality randomised controlled trial (RCT)) and 55 are based on “expert opinion” alone [1]. The lack of evidence is likely due, in part, to the numerous challenges that prevent the conduct of high-quality trials.

First, the critical care population is heterogeneous [4]. However, the metabolic response to injury is not heterogenous [5,6], with individual patient differences in nutrient digestion, absorption, and utilisation variably influenced by a number of factors, including body composition, age, illness/injury severity, co-morbidities, administered medications, and medical interventions such as renal replacement therapy and extracorporeal membrane oxygenation. Despite this, large critical care studies regularly recruit a heterogeneous population with limited ability for adjustment or consideration of the effect that individual patient characteristics have on the interpretation of results. This may occur, in part, for convenience, as it allows for the recruitment of large participant numbers. In addition, it may reflect that clinicians often apply feeding in a universal manner across the patient population; how different patient populations may respond to therapies is unknown.

Second, critical care nutrition trials are difficult, and sometimes impossible, to conduct blindly, particularly those relating to the route (enteral versus parenteral) or mode (intermittent versus continuous) of delivery. While many attempts have been made, very few RCTs have managed to achieve rigorous blinding processes that include investigators, clinical staff, participants, and outcome assessors [7]. Another major challenge of nutrition research in any field is the inability to control individual aspects of nutrition without inadvertently affecting others. For example, adjusting one macronutrient means affecting either calorie delivery or another macronutrient; therefore, it can be difficult to ascertain what is driving the final result.

Patient-centred outcomes that are likely to be influenced by nutrition are long term, potentially subjective, and; hence, difficult to quantify. Pharmaceutical trials exploring blood pressure medication can quantitatively and accurately ascertain the effect on blood pressure (a conveniently and accurately measured known surrogate for survival); however, nutrition outcomes are less tangible, with no identifiable surrogate. Mortality or calorie delivery are most commonly used [8]. An outcome measure likely to be related to nutrition is functional recovery, but the best way to quantify this objectively has yet to be identified. Furthermore, the benefit of nutrition, particularly on outcomes such as survival, is likely to be small and, therefore, difficult to detect [9]. Correspondingly, while core outcome sets exist for other areas of critical care, they do not exist in the field of nutrition. How these aspects have been managed in trial design and evolved over time is discussed in this review (summarised in Table 1), with the timeframes defined as past (pre-2010), present (2010–2020), and future (2020 and beyond).

## 2. Study Design in the Past

### 2.1. Methodology

Early critical care nutrition research was largely observational, initially measuring and documenting patients’ clinical progress and later quantifying aspects of physiology and pathophysiology, such as the measurement of energy expenditure and urinary nitrogen loss. Observational research remains important as it helps us identify, characterise, and quantify clinical problems. Early critical care nutrition studies taught us that the catabolic process of critical illness results in a body mass loss and muscle breakdown, and that this is associated with an excess of nitrogen loss in the urine, indicating protein breakdown [10]. In addition, critically ill patients were shown to have normal or above normal energy expenditure [11], which increased with greater injury severity [12].

In the more recent past (prior to 10 years ago), small single-centre interventional trials were undertaken. These were somewhat limited, given our understanding today, but nevertheless provided preliminary data on which to base subsequent research. In these trials, study methodology was not publicly described prior to analysis or publication, randomisation was not always undertaken and was not necessarily performed optimally, blinding and allocation concealment were not always maintained, outcomes were poorly defined and often not identified prior to data analysis, and statistical analyses were undeveloped compared to current approaches. These limitations were partly due to a lack of understanding of study methodology, as well as an inability to perform large clinical trials due to lack of resources and limited access to appropriately sized patient groups.

### 2.2. Population

Early critical care nutrition trials tended to include specific populations studied at specialist centres or by specialist groups. These included patients with burns [13], traumatic brain injury [14,15,16], or acute respiratory distress syndrome [17]. Convenience samples were frequently used, which likely led to biased results.

### 2.3. Interventions

Prior to the year 2000, interventional trials concentrated on methods of feeding: such as timing (e.g., early versus later initiation [18]); and route of delivery (e.g., gastric versus jejunal [19,20] and enteral nutrition (EN) versus parenteral nutrition (PN) [21,22]). Studies on macronutrients included protein- versus glucose-based PN [23,24,25,26,27], medium- versus long-chain triglycerides [28,29,30,31], and branched chain amino acids [32,33,34]. Immunonutrition was very popular as it was seen to have the potential to blunt the inflammatory response [17,35,36,37,38]. Many initiatives were driven by industry, rather than investigator-initiated [37].

Interventions in the next decade (2000–2010) continued with some of the same themes, but also started to look at different processes for delivering feeding, including the effects of feeding according to evidence-based algorithms [39,40] and continuous versus bolus feeding [41]. Immunonutrition continued to be popular, but some investigators started to look at different lipids, as well as at other nutritional additives such as probiotics [42,43].

### 2.4. Outcomes

Physiologically-based, short-term surrogate outcomes that had not yet been linked to important clinical outcomes were frequently used. These can be categorised into nutritional outcomes (e.g., calorie delivery, nitrogen balance, pre-albumin, and fibronectin concentrations), markers of inflammation (e.g., T cell numbers and C-reactive protein levels), and metabolic indices, (e.g., catecholamine levels). While it was assumed that an improvement in one of these surrogate outcomes may have a beneficial effect on survival, muscle mass, or clinical outcomes, this had not been confirmed. Later trials measured clinical outcomes such as infections, duration of ventilation, length of stay in the ICU, and survival, but many were not adequately powered to detect an effect.

### 2.5. Interpretation of Research and Translation into Clinical Practice

A further limitation was the way medical evidence was interpreted and translated into clinical practice. For example, small observational studies were able to demonstrate that high calorie delivery (3000–4000 calories/day) from glucose and protein reduced urinary nitrogen loss. It was consequently assumed that this would also reduce muscle mass loss, and thus the practise was widely introduced without an understanding of whether this had positive or deleterious effects on other parameters, particularly patient-centred outcomes such as survival or recovery. Another widespread assumption was that energy delivery should match energy expenditure. With this in mind, and because the measurement of energy expenditure was not easy in a clinical setting, multiple equations were derived to calculate energy expenditure, none of which proved reliably accurate in subsequent comparative studies. Progress in understanding of trial methodology and statistical analysis has occurred alongside progress in medical therapies, and this has formed the basis for advances in research processes.

## 3. Study Design in the Present

Over the last 10 years, research into critical care nutrition has become a focus for large research groups with international reputations for well-conducted, high-quality trials. Recognising the pitfalls of previous work and the predominance of observational evidence or underpowered studies in guiding practice, it became obvious that these smaller, less robust studies generated hypotheses that needed to be tested in a more rigorous way. In addition, trialists brought lessons from the conduct of high-quality clinical research in other areas to the field of critical care nutrition.

### 3.1. Methodology

In the past 10 years, at least nine phase III trials investigating nutrition intervention in critical care have been published by leading investigators in the United Kingdom, Australia and New Zealand, Saudi Arabia, the United States, Canada, and Europe [7,44,45,46,47,48,49,50,51]. The key features of these trials are the large sample sizes (often in the thousands), the methodological quality (specifically pilot work, pre-specified protocols, statistical analysis plans, blinding, and randomisation), and rigorous trial management procedures including data safety monitoring committees and interim analyses with a priori defined stopping rules. 

### 3.2. Population

The majority of recently conducted trials have focused on a heterogenous critically ill population, as studies involving specific populations are challenging in terms of recruitment and are hence underpowered. Moreover, subgroup analyses of the larger RCTs, while underpowered to give a definitive answer for the intervention, have been conducted to elucidate populations that may benefit from further study. In the most recent trials, a focus on specific populations with more carefully defined inclusion criteria is starting to emerge; however, this is still an area necessitating greater focus [46,52], and the effect on generalisability requires consideration.

### 3.3. Interventions

In keeping with the inclusion of heterogenous populations, the majority of recently conducted trials have addressed general questions regarding critical care nutrition practices (e.g., calorie targets and EN versus PN). An issue with this work is the duration of the interventions, being delivered in the acute early phase of critical illness for around 4–7 days in the majority of cases, which may reflect the brief period patients receive artificial nutrition support in the ICU compared to the overall hospital admission. This is likely associated with the populations being studied (general ICU patients), for whom the average length of stay is short (and decreasing year on year in developed countries). This has implications for an intervention such as nutrition, where, unlike that of a drug, the impact is not immediate; a patient’s “nutrition journey” does not end at ICU discharge, and a nutritional intervention applied for just a few days may not be expected to have an influence on clinical outcomes. Similarly, trials have largely treated nutrition like a pharmacological intervention or fluid, with little consideration for its complexity. What these studies have provided is high-quality evidence regarding the impact of short-term nutrition interventions in heterogenous critically ill patients for the early part of their critical illness (i.e., the time they are fed in the ICU). This provides a crucial platform for future work.

### 3.4. Outcomes

As is the case for study methodology, concepts from other areas of critical care medicine have been utilised in the nutrition field, with the recent inclusion of more robust clinically focused outcomes. These outcomes are ideally objective (e.g., mortality) and important to patients, clinicians, and researchers alike. However, the plausibility of whether short-term nutrition interventions are likely to influence a much later outcome should be considered. Furthermore, the effect that a nutrition intervention may have on a clinical outcome such as mortality is likely to be small, making the required sample size large, and meaning that most previous RCTs (even if well designed and conducted) may have been underpowered for the primary outcome [53]. More recently, trials have used markers of muscle change as a primary outcome, a sign that investigators are starting to consider the biological plausibility of the intervention on the outcome [54,55,56]. However, it must be acknowledged that the trials that have included these alternate outcomes are relatively small, a link between muscle change and clinically important outcomes is required, and the lack of standardised procedures used to conduct these measurements is likely to effect the appropriate interpretation of results [57].

## 4. Study Design in the Future

Critical care nutrition trial design is evolving rapidly, with innovative study designs, greater consideration of duration and timing of, and individual responses to, interventions, and incorporation of patient-centred outcomes.

### 4.1. Methodology

As survival rates in ICUs improve, the ability for a single intervention to demonstrate a beneficial effect on mortality will likely require a very large sample size (i.e., tens of thousands of participants) [58]. Nutrition interventions may be beneficial, but the measurable effects are likely to be small because of the numerous factors, both patient- and treatment-related, affecting outcomes in critically ill patients. One of the largest critical care nutrition studies to date, which randomised just under 4000 patients across 46 ICUs, was powered to detect a 3.8%–4.3% mortality difference and cost upwards of 4.5 million dollars (Australian) to conduct. Even smaller survival benefits would be useful to determine, but with a parallel RCT design in a heterogenous population, this would require very large sample sizes, and hence would likely be logistically or financially unfeasible. Trial design needs to adapt accordingly, and future critical care nutrition research is likely to see more innovative and sophisticated methodology. The use of registry data or cluster randomisation may facilitate greater patient numbers [59]; however, this comes with limitations in relation to the restricted data points and availability of key nutrition data [60]. The first multi-centre randomised critical care nutrition trial to use registry data (EFFORT trial) is currently recruiting study subjects to ascertain the influence of higher protein doses on mortality [61]. Other study methodologies that may lend themselves to nutrition interventions are adaptive or Bayesian-style designs that allow iterative changes in response to therapy benefits that arise during the trial [62,63]. While these are currently being explored in other critical care interventions [64], identification of an important short-term outcome likely to be influenced by nutrition is needed prior to consideration of these types of methodologies for critical care nutrition.

Large randomised trials are unlikely to demonstrate positive results if they are not prefaced by rigorous preliminary research; thus, large trials should be preceded by detailed mechanistic work embedded within the research programme rather than a siloed research stream [65,66]. As trials become more complex, the use of artificial intelligence [67], as well as the involvement of experienced clinician-researchers and biostatisticians, will likely be integral to study design and data interpretation. In addition, greater transparency of study methodology should include a pre-published protocol and statistical analysis plan, trial registration, and clear reporting of both results and data analysis, including those for post-hoc analyses. Access to open-source data after publication should also be considered to allow for analysis by an independent statistician to assess reproducibility.

### 4.2. Population

Future critical care nutrition research is likely to place a greater focus on specific clinical populations, with greater consideration of individual patient characteristics to enable personalised or individualised therapy and to account for factors such as pre-morbid nutrition status, body composition, and stage of disease. A minimum common dataset of patient population characteristics to be reported may support the advancement of this, as too may the use of metabolic biomarkers to determine those patients most likely to benefit from nutrition support [68,69]. Though nutrition risk scores have been developed, they have been poorly validated, are often inaccurate, and may reflect injury severity more than nutrition risk [70], which may reduce the number of patients needing to receive the intervention in order to see benefit.

Furthermore, as medical therapy advances, so too must nutrition therapy. Management strategies for conditions such as acute kidney injury, delirium, and burns may affect nutrition requirements or delivery, and hence nutrition support specific to these conditions requires exploration. There is also a growing population of patients that are either never mechanically ventilated or ventilated for a short period of their ICU stay only, yet international nutrition guidelines provide limited recommendations specific for this population and; hence, the nutritional needs of this population must be ascertained.

### 4.3. Interventions

To date, critical care nutrition interventions have been provided while the patient remains within the ICU setting [71]. Yet this reflects just a short phase of the patient’s hospitalisation, and post-ICU nutrition intake has recently been shown to be poor [72,73]. We are starting to see more focus on what happens to critically ill patients after ICU discharge (INTENT trial, clinicaltrials.gov: NCT03292237). In addition, it is important that the timing of an intervention takes into account the stage of illness and metabolic state rather than simple chronology, and includes reference to identifying the “metabolic switch” for ICU patients [69], as well as recognising the patient’s recovery trajectory in order to intervene appropriately [74]. This has been identified in the general ICU literature stating that the changing pathophysiology of a disease within the same patient needs to be considered [58]. A key aspect of this is the careful documentation and appropriate analysis of the duration during which the target intervention was received, taking into consideration the progressive delivery of nutrition in order to reach goal rates early while in the ICU, as well as weaning from nutritional support, a concept well described previously [75,76].

As nutrition knowledge progresses, we may start to see more individualised treatments, such as patient-specific nutrition formulations (e.g., chemotherapy specifically blended for the best outcome), that take into consideration individual requirements for fluid, volume, and macro- and micro-nutrients, and that change over time as individual needs evolve [77]. Bundled or synergistic therapies should also be considered, such as nutrition and physical therapy [69,78].

### 4.4. Outcomes

To date, large phase III RCTs in critically ill patients have used mortality as the primary outcome [9,79], yet few have reported benefit from general ICU studies [80] or nutrition-specific interventions [8]. In the next decade, we are likely to see greater emphasis placed on what is important to patients, (e.g., functional, social, and psychological recovery), increased consumer engagement [81], and incorporation of co-design that includes not only clinicians/researchers, but also patients, in the trial design [82]. The use of technology such as smartphones or wearable devices will also allow greater analysis of objective outcome measures. The inclusion of body composition outcomes has been highlighted in international research agenda [83], and technology such as continuous glucose monitors that allow minute-by-minute data points affords immediate responses to nutrition therapies in need of assessment.

## 5. Conclusions

In the last decade critical care nutrition research has evolved rapidly. Early physiological studies, observational data, and small comparative trials have made way for large, definitive, rigorously conducted RCTs. Each trial has added incremental evidence that has made clinical practice what it is today. A number of research agendas exist that provide tangible guidance on the future research questions considered of utmost importance in the critical care nutrition field, as well as methodological challenges to be considered by clinical researchers and trialists [6,69]. Future trials will have new challenges. They must embrace technology and intelligent study design in order to cost-effectively recruit larger numbers of patients or to target those most likely to benefit in the critical phase of their hospital journey. Critical care nutrition research will continue to advance, providing clinicians and researchers alike with a greater understanding of the nutrition practices that will ultimately deliver benefit for their patients. 

## Figures and Tables

**Table 1:** Summary of critical care nutrition study design in the past, present, and future.

	Past	Present	Future
Study methodology	Small physiological, observational, and single-centre comparative trials; poorly defined and underdeveloped processes	Large phase III trials (usually in the 1000s); a priori defined protocols, outcomes, and interim analyses	Sophisticated statistical techniques; larger sample sizes (tens of thousands); adaptive trial designs
Population	Small, specific populations often from specialist centres	General, heterogenic populations	Homogenous; selected based on anticipated response to nutrition intervention (e.g., malnourished); includes non-invasively ventilated cohorts
Intervention	Calories; early vs. late initiation; route of delivery e.g., gastric vs. jejunal, EN vs. PN, protein- vs. glucose-based PN, medium- vs. long-chain triglycerides and branched chain amino acids; immunonutrition	General interventions for general questions; focused only on the period in ICU	Synergistic; patient-specific; based on mechanisms; extension of interventions beyond ICU discharge considering illness trajectory
Outcomes	Calorie delivery; nitrogen balance; incidence of infection; mortality (but underpowered to show an effect in the latter)	Robust clinical outcomes such as mortality	Patient recovery; functional outcomes; valid surrogate markers

Abbreviations: EN = enteral nutrition; ICU = intensive care unit; PN = parenteral nutrition.
